# DBGC: A Database of Human Gastric Cancer

**DOI:** 10.1371/journal.pone.0142591

**Published:** 2015-11-13

**Authors:** Chao Wang, Jun Zhang, Mingdeng Cai, Zhenggang Zhu, Wenjie Gu, Yingyan Yu, Xiaoyan Zhang

**Affiliations:** 1 School of Life Science and Technology, Tongji University, 1239 Siping Road, Shanghai, China; 2 Shanghai Ruijin Hospital, Shanghai Institute of Digestive Surgery and Shanghai Key Laboratory of Gastric Neoplasms, Affiliated to Shanghai Jiaotong University, School of Medicine, Shanghai, China; 3 Collaborative Innovation Center of Systems Biomedicine, Shanghai Jiao Tong University, 200025, Shanghai, China; Baylor College of Medicine, UNITED STATES

## Abstract

The Database of Human Gastric Cancer (DBGC) is a comprehensive database that integrates various human gastric cancer-related data resources. Human gastric cancer-related transcriptomics projects, proteomics projects, mutations, biomarkers and drug-sensitive genes from different sources were collected and unified in this database. Moreover, epidemiological statistics of gastric cancer patients in China and clinicopathological information annotated with gastric cancer cases were also integrated into the DBGC. We believe that this database will greatly facilitate research regarding human gastric cancer in many fields. DBGC is freely available at http://bminfor.tongji.edu.cn/dbgc/index.do

## Introduction

As one of the most common cancers, gastric cancer has the third highest lethality and fourth highest morbidity of all cancers worldwide [1]. According to the GloboCan statistics in 2012, new gastric cancer cases numbered almost one million (952,000), and more than 700,000 deaths were caused by gastric cancer; almost half of these patients came from China (405,000 new cases and 325,000 deaths) [1, 2]. Although both the lethality and morbidity of gastric cancer have decreased in recent years, the 5-year survival rate remains quite low [3]. Therefore, gastric cancer will remain one of the most difficult challenges for researchers and physicians for a long time [4].

Researchers worldwide have completed many genomics, transcriptomics, proteomics, and epidemiological investigations and clinical trials regarding the pathogenesis and therapies of gastric cancer [5–10]. These investigations have generated vast amounts of data relevant to gastric cancer, and the speed of these investigations is accelerating with the rapid growth of cancer knowledge, decreased costs of detection and computation, and spread of the Internet [11]. These data contain important information for investigating and curing gastric cancer. However, due to the limited background knowledge of clinicians and fundamental researchers, the potential of these data cannot be fully developed. New technologies and research methods still require development; however, low efficiency in managing data is a primary limitation of this development [12]. Due to the long-term accumulation of decentralized research, these data and their formats only satisfy individual needs, lacking integration and standardization and resulting in the diversification, isomerization, and dissection of cancer data [13, 14].

At present, abundant clinical and fundamental studies regarding gastric cancer are planned or in progress. Various types of data are stored in different database systems [13], without sharing or communication. Thus, strongly correlated information remains isolated, in what are called “information islands”. On the one hand, data dissection increases the difficulty of data mining, while on the other hand, it prevents clinicians from making full use of the outcomes of fundamental research to develop clinical trials and applications and keeps fundamental researchers from performing efficient exploratory studies that reference clinically relevant information [15].

In this situation, retrieving comprehensive information on gastric cancer is not an easy task, and portions of these data may disappear in the ocean of the Internet, which would be very unfortunate.

This research took advantage of resources from the Internet and publications from the Chinese Center for Disease Control and Prevention (CDC) and Gastric Cancer Center for Diagnosis and Treatment, Key Laboratory of Gastric Neoplasms in Shanghai. This study systematically collected various types of gastric cancer-related data, integrated these data resources after filtration and standardization, and finally formed the first comprehensive knowledge base for analyzing gastric cancer.

## Materials and Methods

### Data Resources

The Database of Human Gastric Cancer (DBGC) has integrated the following gastric cancer-related resources:

1. Epidemiological statistics of gastric cancer patients in China from CDC publications
2. Clinicopathological information about gastric cancer tissue after surgical resection from patients diagnosed at Shanghai Ruijin Hospital
3. Molecular biological data on gastric cancer from public online resources (including gastric cancer-related mutations, biomarkers, drug-sensitive genes, transcriptomics projects and corresponding differentially expressed genes, and proteomics projects and corresponding differentially expressed proteins)
4. Raw research data from the Shanghai Institute of Digestive Surgery and Shanghai Key Laboratory of Gastric Neoplasms

### Data Collection

#### 1) Epidemiological statistics of gastric cancer patients in China

The CDC has had an established cancer reporting system for many years and has accumulated abundant epidemiological information on cancer patients in China. The epidemiological statistics of gastric cancer, including case number, death number, incidence rate (crude rate, age-adjusted rate and cumulative rate), mortality rate (crude rate, age-adjusted rate and cumulative rate), and incidence (or mortality) distribution by age group were extracted manually from CDC publications. DBGC 1.0 covers all epidemiological statistics for all typical regions of China from the years 2004 to 2009, and additional statistics will be included in the upgraded version.

#### 2) Clinicopathological information about gastric cancer tissue

Clinicopathological information was provided by Shanghai Ruijin Hospital. The classification and staging methods generally used for gastric cancer diagnosis were annotated using gastric cancer cases diagnosed at Ruijin Hospital. Typical gastric cancer tissues of different stages and types were selected from a gastric cancer biobank that we have maintained for years. All patient information was anonymized and de-identified before our analysis.

#### 3) Molecular biological data on gastric cancer from public online resources

Molecular biological data were extracted and curated from online resources. Transcriptomics data were collected from the GEO database (http://www.ncbi.nlm.nih.gov/geo/) and EBI database (http://www.ebi.ac.uk/). Proteomics data were extracted from the published literature through manual reading and standardization [16, 17]. Mutation data were collected from the dbVar database (http://www.ncbi.nlm.nih.gov/dbvar/), OMIM database (http://www.ncbi.nlm.nih.gov/omim/), HGMD database (http://www.hgmd.org/), and published literature [18, 19]. All biomarker data were extracted from published literature [20, 21]. Drug-related genes were extracted from the PharmGKB database (http://www.pharmgkb.org/), CancerDR database (http://crdd.osdd.net/raghava/cancerdr/) and published literature [22, 23]. We designed detailed extraction standards for each type of molecular biological data resource, and every data collection procedure had to follow these standards to ensure data coherency. The detailed collection procedure is provided below:

Transcriptomics data:

1. Search the GEO database using the following keywords:(“stomach neoplasms” [MeSH Terms] OR “stomach cancer” [All Fields]) AND "Homo sapiens" [porgn].
2. Filter the results manually, and select the publications related to human gastric cancer for subsequent information extraction.
3. Classify the programs by sample size and sample type.
4. Extract publication information (title, publication time, experiment type, tissue type, sample quantity, sample description, sample of experiment, sample of control, platform, GSE ID, GSM IDs, download links, and literature citation) manually, referring to MIAME (Minimum Information About a Microarray Experiment).
5. Pre-process raw data (series matrix files in the GEO database) using Perl to eliminate the differences from diverse platforms.
6. Extract differentially expressed genes using R language.

Proteomics data:

1. Search raw data in PubMed using the following keywords: (“proteomics” [MeSH Terms] OR “proteomics” [All Fields]) AND (“stomach neoplasms” [MeSH Terms] OR (“stomach” [All Fields] AND “neoplasms” [All Fields]) OR “stomach neoplasms” [All Fields] OR (“gastric” [All Fields] AND “cancer” [All Fields]) OR “gastric cancer” [All Fields]).
2. Filter the results manually, and select the proteomics publications related to human gastric cancer for subsequent information extraction.
3. Use these papers as seed literature and filter the references again.
4. Classify the publications by sample size and sample type.
5. Manually read papers and extract publication information (title, publication time, sample quantity, sample experiment, sample control, sample description, technology method used, fold change, up-regulated protein quantity, down-regulated protein quantity, and reference) and corresponding up-regulated proteins and down-regulated proteins (based on conclusions of the authors).

Mutation data:

1. Search in the OMIM, HGMD and dbVar databases using the keywords “gastric cancer” and extract mutation information (gene, mutation type, description of cDNA, description of full AA, description of AA, and reference).
2. Search in PubMed using the following keywords: (“mutation” [MeSH Terms] OR “mutation” [All Fields]) AND (“stomach neoplasms” [MeSH Terms] OR (“stomach” [All Fields] AND “neoplasms” [All Fields]) OR “stomach neoplasms” [All Fields] OR (“gastric” [All Fields] AND “cancer” [All Fields]) OR “gastric cancer” [All Fields]).
3. Filter the results manually, and select the papers related to human gastric cancer for subsequent information extraction.
4. Take these papers as seed literature and filter the references again.
5. Read these papers and extract mutation information manually (gene, mutation type, description of cDNA, description of full AA, description of AA, and reference).
6. Remove duplicate data from the four sources.

Biomarker data:

1. Search in PubMed using the following keywords:
  (“biological markers” [MeSH Terms] OR (“biological” [All Fields] AND “markers” [All Fields]) OR “biological markers” [All Fields] OR “biomarker” [All Fields]) AND (“stomach neoplasms” [MeSH Terms] OR (“stomach” [All Fields] AND “neoplasms” [All Fields]) OR “stomach neoplasms” [All Fields] OR (“gastric” [All Fields] AND “cancer” [All Fields]) OR “gastric cancer” [All Fields]).
2. Filter the results manually, and select the papers related to human gastric cancer for subsequent information extraction.
3. Take these papers as seed literature and filter the references again.
4. Read these papers and extract mutation information manually (biomarker name, full name, type, stage, description, mechanism, sensitivity, specificity, and reference).
5. Classify the biomarkers by biomarker type, stage, specificity and sensitivity.

Drug-sensitive data:

1. Search in PharmGKB using the keywords “gastric cancer” and manually extract drug-sensitive information (drug name, gene name, gene type, mechanism, and reference).
2. Search in PubMed using the following keywords:“resistance” [All Fields] AND (“stomach neoplasms” [MeSH Terms] OR (“stomach” [All Fields] AND “neoplasms” [All Fields]) OR “stomach neoplasms” [All Fields] OR (“gastric” [All Fields] AND “cancer” [All Fields]) OR “gastric cancer” [All Fields])
3. Filter the results manually, and select the papers related to human gastric cancer drug resistance for subsequent information extraction.
4. Take these papers as seed literature and filter the references again.
5. Summarize the 19 drugs generally used for the clinical treatment of gastric cancer (5-fluorouridine, camptothecin, carboplatin, cisplatin, docetaxel, doxorubicin, doxorubicin hydrochloride, epirubicin, etoposide, fluorouracil, irinotecan, leucovorin, mitomycin c, oxaliplatin, paclitaxel, tamoxifen, trastuzumab, vinblastine, and vincristine).
  Taking “cisplatin” as an example, search in PubMed using keywords:
  (“cisplatin” [MeSH Terms] OR “cisplatin” [All Fields]) AND “resistance” [All Fields] AND (“stomach neoplasms” [MeSH Terms] OR (“stomach” [All Fields] AND “neoplasms” [All Fields]) OR “stomach neoplasms” [All Fields] OR (“gastric” [All Fields] AND “cancer” [All Fields]) OR “gastric cancer” [All Fields]).
6. Filter the results manually, and select the papers related to human gastric cancer drug resistance for subsequent information extraction.
7. Take these papers as seed literature and filter the references again.
8. Read these papers and extract drug-sensitive information manually (drug name, gene name, gene type, mechanism, and reference).

We annotated all genes and drugs in this database to help users better understand and use these data resources. The genes are annotated according to NCBI (http://www.ncbi.nlm.nih.gov), HGNC (http://www.genenames.org/), Ensembl (http://feb2014.archive.ensembl.org/) and Gene Cards (http://www.genecards.org/). The drugs are annotated according to DrugBank (http://www.drugbank.ca/).

Moreover, mutations detected in the TCGA project were also included to annotate genes in the DBGC. Users can find all mutations of a certain gene detected in the TCGA project. These mutations were processed by ICGC (https://dcc.icgc.org) based on TCGA data and referenced by each mutation in the DBGC.

In addition, several gastric cancer-related fundamental research projects have been conducted by our research team. Project descriptions and raw data are provided in the DBGC for download and further analysis.

### Database Construction

The DBGC is a relational database with a MySQL data layer. A user-friendly interface was designed to organize and display data resources using HTML and JavaScript. The interaction between the data layer and the web interface was completed using the Java EE Platform.

## Results and Discussion

### Database Description

**Fig 1:**
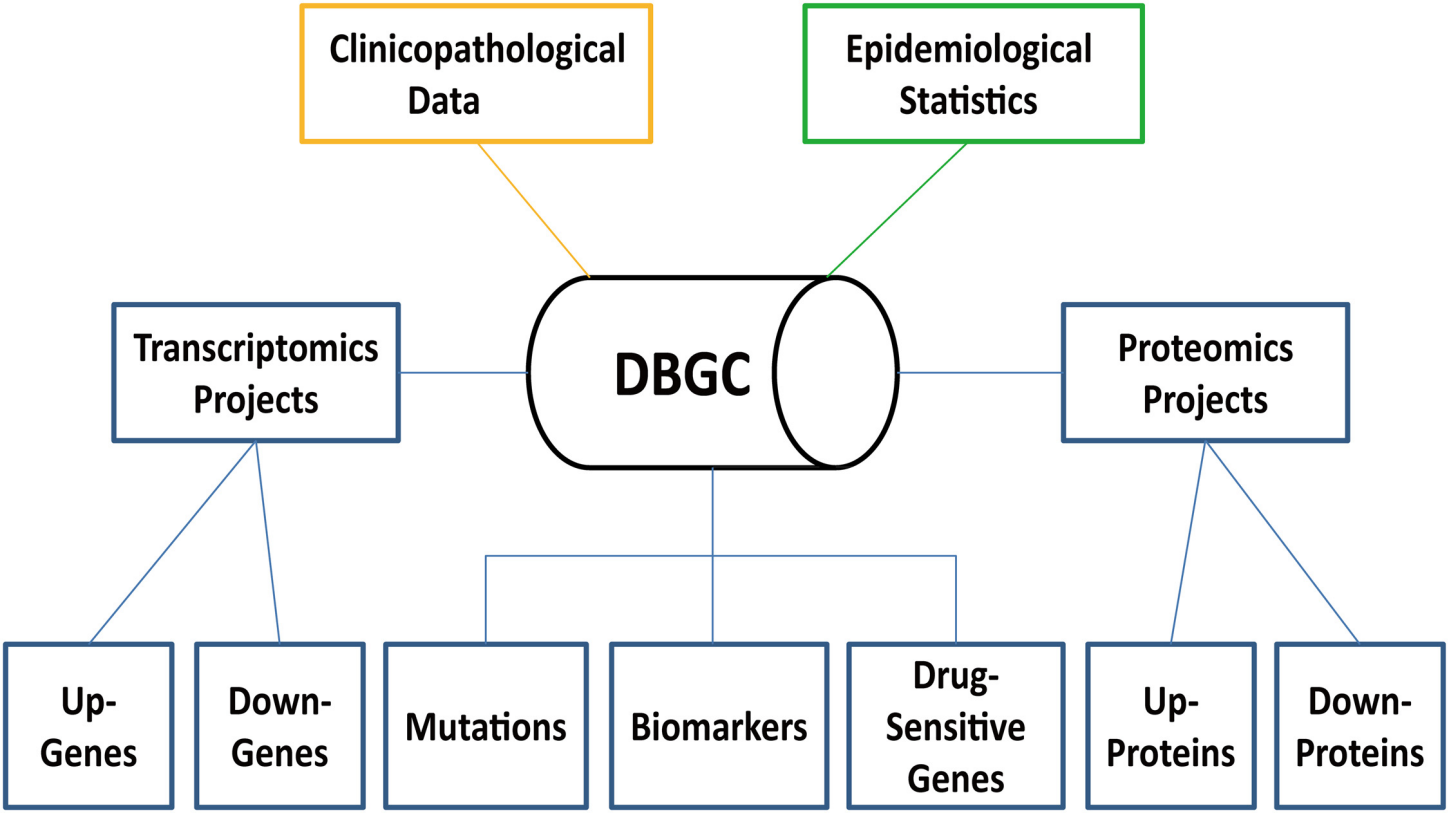
Schematic architecture of the DBGC.

This database consists primarily of three longitudinal data systems, epidemiological, clinicopathological and molecular biological data (Fig 1). The molecular biological data consist of gastric cancer-related transcriptomics, proteomics, mutation, biomarker and drug-sensitive gene data. The overall statistics of these data are listed in Table 1. Along with the epidemiological statistics of gastric cancer patients in China and the clinicopathological information annotated with gastric cancer cases, all these data were extracted from public databases, publications and published literature.

**Table 1:** Primary statistics of molecular biological data in the DBGC.

Data resource type	Description	Statistics
Transcriptomics data		
	Transcriptomics projects	121
	Samples	5629
	Up-regulated genes	3463
	Down-regulated genes	3380
Proteomics data		
	Proteomics projects	47
	Up-regulated proteins	1513
	Down-regulated proteins	808
Mutation data		
	Mutations	850
	Genes	97
	TCGA Mutations	1049822
Drug-sensitive data		
	Genes	132
	Drugs	19
Biomarker data		
	Biomarkers	221

### Database Interfaces

**Fig 2:**
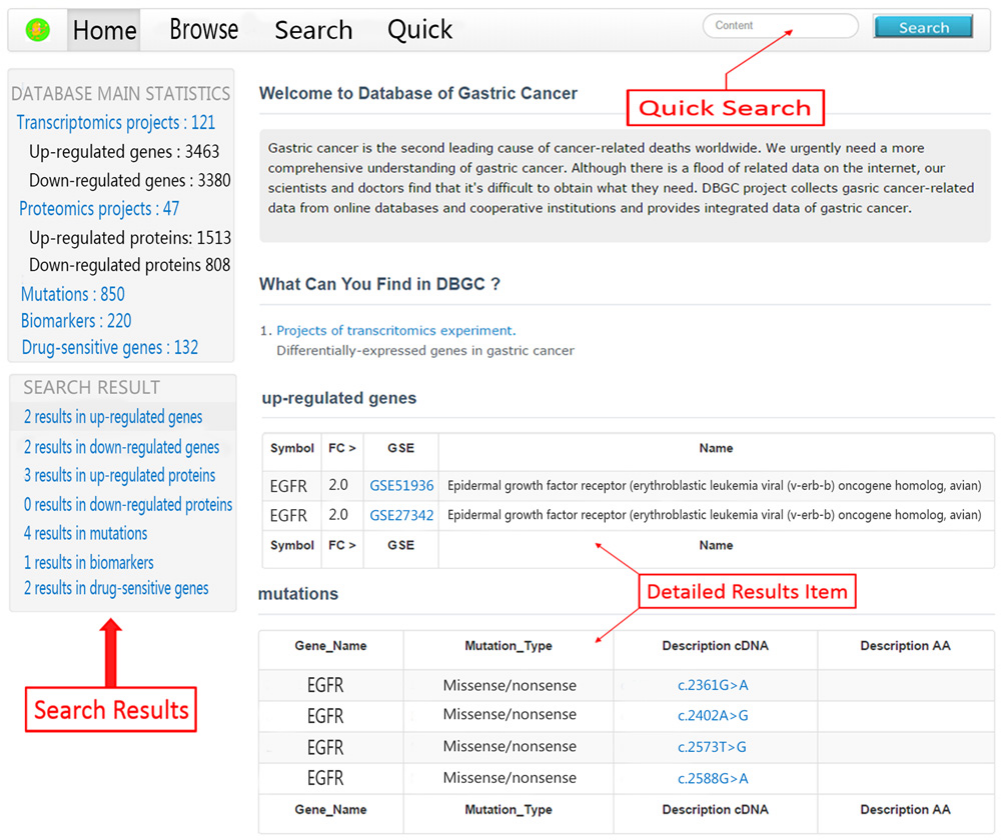
Quick search function module.

**Fig 3:**
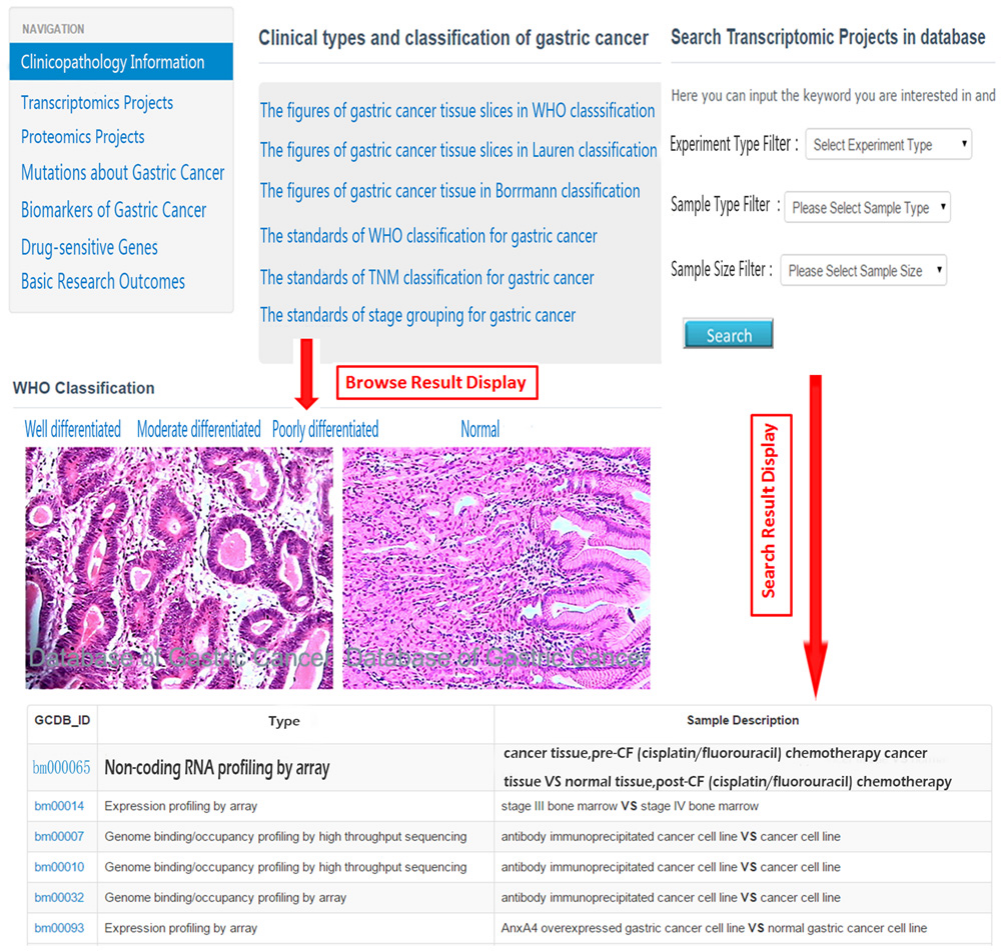
Browse function module and search function module.

**Fig 4:**
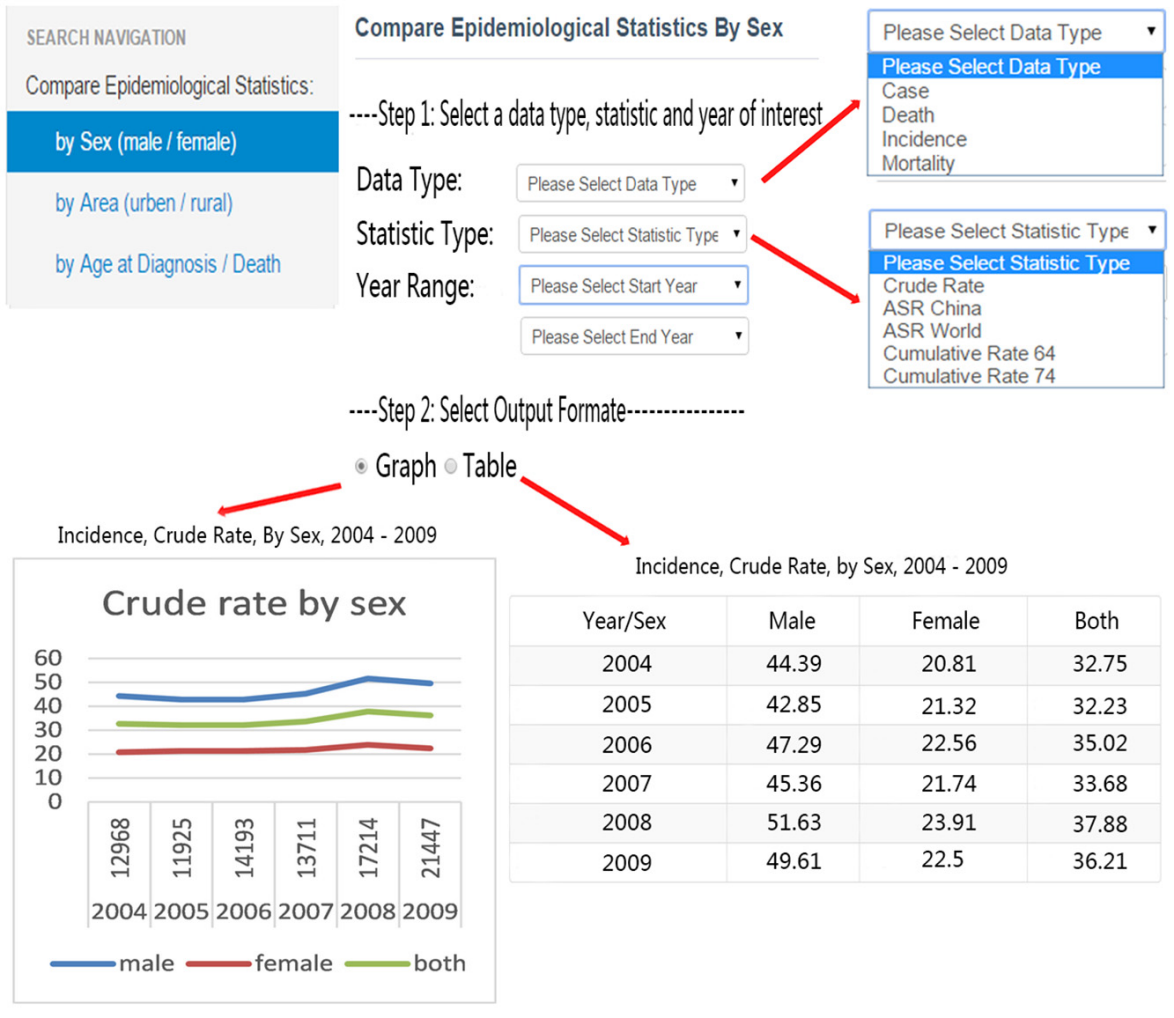
Statistical function module for epidemiological data.

1) Quick Search (Fig 2). The quick search function module makes identifying the role of a gene or protein in gastric cancer possible via inputting keywords into the search box located at the navigation bar. The search result will tell you whether the gene or protein is differentially expressed in any transcriptomics projects or proteomics projects and whether it has been identified as a biomarker for gastric cancer or a drug-sensitive gene. Moreover, if the gene has any mutation that is related to gastric cancer, a detailed list will be displayed on the results page. For example, using “EGFR” as a keyword, we can conclude that it was identified as an up-regulated gene in GSE51936 and GSE27342 and as a down-regulated gene in GSE29630. The corresponding protein of the gene EGFR was identified as an up-regulated protein in 3 proteomics projects (PubMed Ids: 23161554, 24263233 and 24722433). EGFR has been reported as a prognosis factor of gastric cancer and is related to drug resistance to irinotecan, which is a commonly used drug for the treatment of gastric cancer. Four mutations of EGFR related to gastric cancer have been reported (c.2361G>A, c.2402A>G, c.2573T>G, c.2588G>A).

2) Browse and Search (Fig 3). Using the navigation, users can click corresponding items to browse the data resources provided in the DBGC. Detailed information will be listed below. We have also established several search criteria for each type of data resource through which all data items that fulfill the conditions will be displayed.

3) Our database differs from other online resources because of the inclusion of epidemiological statistics of gastric cancer patients in China. Users can compare statistics by sex (male and female), area (urban and rural), and age at diagnosis or death. Case number, death number, incidence rate, and mortality rate in a selected year range can be displayed in both graph and table format (Fig 4).

## Discussion

Gastric cancer is a leading cancer worldwide in both mortality and morbidity. Higher incidence and mortality of gastric cancer are observed in Asian regions, particularly in China. The epidemiological statistical data of gastric cancer in this database were obtained primarily from publications of the CDC, which has been engaged in malignant tumor studies for several decades and has established comprehensive archives of malignant tumor patients in China. These data have played an important role in promoting cancer prevention and health policymaking in China [24–26]. Through retrieving the gastric cancer epidemiology data in this database, researchers and clinicians can quickly determine the epidemiological trends of gastric cancer in China.

Gastric cancer-related mutations, biomarkers, drug-sensitive genes, transcriptomics projects and corresponding differentially expressed genes, and proteomics experiments and corresponding differentially expressed proteins were manually collected from online databases and published literature. The quick search function provided by the DBGC enables researchers to identify the role of a gene or protein in gastric cancer. These differentially expressed genes and proteins contain abundant important information about gastric cancer, and many analytical studies could be conducted using them.

Our research team has been engaged in gastric cancer research for many years and has accumulated considerable experience in tumor epidemiological research, gastric cancer clinicopathology and biomarker research, biobank building, molecular biological mechanism research, bioinformatics analysis and large-scale database construction [27–30]. To provide inquiry and analysis tools that are more convenient and practical for gastric cancer researchers, we constructed this database. The current version is 1.0. Because the large amounts of data generated by different experimental platforms in different fields are greatly dispersed and heterogeneous, some useful information may have been missed in our process of data collection. We will continue retrieving these data and updating the latest data for a long time to ensure the timeliness and completeness of the data. In the next version, we intend to cover the newest human gastric cancer-related mutations, biomarkers and drug-sensitive genes. Transcriptomics data will be the emphasis of the next version, in which all transcriptomics projects will be re-analyzed to extract differentially expressed genes at different fold change values. Thus, users could query whether a certain gene is differentially expressed through configuring the sample type and fold change value.

## Conclusion

The database described in this article, the DBGC, is a comprehensive and web-accessible database of human gastric cancer. This database has integrated a variety of data resources related to gastric cancer and provided several easy-to-use web-based functional modules. We believe that the DBGC will be an important tool for gastric cancer clinicians, tumor fundamental research scientists, cancer genome researchers, government health policymakers, and gastric cancer patients.
